# Micro-Structural Brain Alterations in Aviremic HIV+ Patients with Minor Neurocognitive Disorders: A Multi-Contrast Study at High Field

**DOI:** 10.1371/journal.pone.0072547

**Published:** 2013-09-10

**Authors:** Cristina Granziera, Alessandro Daducci, Samanta Simioni, Matthias Cavassini, Alexis Roche, Djalel Meskaldji, Tobias Kober, Melanie Metral, Alexandra Calmy, Gunther Helms, Bernard Hirschel, François Lazeyras, Reto Meuli, Gunnar Krueger, Renaud A. Du Pasquier

**Affiliations:** 1 Department of Clinical Neurosciences, Neuroimmunology Unit, Centre Hospitalier Universitaire Vaudois and University of Lausanne, Lausanne, Vaud, Switzerland; 2 Department of Clinical Neurosciences, Laboratoire de Recherche En Neuroimagerie (LREN), Centre Hospitalier Universitaire Vaudois and University of Lausanne, Lausanne, Vaud, Switzerland; 3 Advanced Clinical Imaging Technology, Centre d’imagerie biomédical, École Polytechnique Fédérale de Lausanne, Lausanne, Vaud, Switzerland; 4 Signal Processing Laboratory (LTS5), École Polytechnique Fédérale de Lausanne, Lausanne, Vaud, Switzerland; 5 Department of Infectious Diseases, Centre Hospitalier Universitaire Vaudois and University of Lausanne, Lausanne, Vaud, Switzerland; 6 Department of Clinical Neurosciences, Neuropsychology Unit, Hôpitaux Universitaires de *Genève*, Geneva, Switzerland; 7 Department of Infectious diseases, Hôpitaux Universitaires de *Genève*, Geneva, Switzerland; 8 MR-Forschung in der Neurologie und Psychiatrie, Georg-August-Universität Göttingen, Germany; 9 Department of Radiology, Hôpitaux Universitaires de *Genève*, Geneva, Switzerland; 10 Department of Radiology, Centre Hospitalier Universitaire Vaudois and University of Lausanne, Lausanne, Vaud, Switzerland; 11 Healthcare, Siemens Schweiz AG, Renens, Vaud, Switzerland; University of Manchester, United Kingdom

## Abstract

**Objective:**

Mild neurocognitive disorders (MND) affect a subset of HIV+ patients under effective combination antiretroviral therapy (cART). In this study, we used an innovative multi-contrast magnetic resonance imaging (MRI) approach at high-field to assess the presence of micro-structural brain alterations in MND+ patients.

**Methods:**

We enrolled 17 MND+ and 19 MND− patients with undetectable HIV-1 RNA and 19 healthy controls (HC). MRI acquisitions at 3T included: MP2RAGE for T1 relaxation times, Magnetization Transfer (MT), T2* and Susceptibility Weighted Imaging (SWI) to probe micro-structural integrity and iron deposition in the brain. Statistical analysis used permutation-based tests and correction for family-wise error rate. Multiple regression analysis was performed between MRI data and (i) neuropsychological results (ii) HIV infection characteristics. A linear discriminant analysis (LDA) based on MRI data was performed between MND+ and MND− patients and cross-validated with a leave-one-out test.

**Results:**

Our data revealed loss of structural integrity and micro-oedema in MND+ compared to HC in the global white and cortical gray matter, as well as in the thalamus and basal ganglia. Multiple regression analysis showed a significant influence of sub-cortical nuclei alterations on the executive index of MND+ patients (p = 0.04 he and R^2^ = 95.2). The LDA distinguished MND+ and MND− patients with a classification quality of 73% after cross-validation.

**Conclusion:**

Our study shows micro-structural brain tissue alterations in MND+ patients under effective therapy and suggests that multi-contrast MRI at high field is a powerful approach to discriminate between HIV+ patients on cART with and without mild neurocognitive deficits.

## Introduction

It has been suggested that HIV encephalitis is the neuropathological substrate of cognitive disorders [1]. Although HIV does not directly infect neurons or oligodendrocytes, this virus can trigger an inflammatory response with release of cytokines, chemokines, and neurotoxic HIV viral proteins (e.g. gp120) [1], [2], leading to inflammatory infiltrates, as well as myelin and neuronal loss [3], [4]. Since the seeding of HIV in the brain occurs early after infection [5], it is possible that HIV-triggered neuro-inflammatory changes occur in the early stages of the disease. Furthermore, combination antiretroviral therapy (cART) may not be sufficient to prevent neuro-inflammatory damages triggered by HIV since some anti-retroviral drugs have a poor rate of penetration-effectiveness into the central nervous system (CNS) [6]. On the other hand, some cART compounds with good penetration might be neurotoxic and provoke cognitive disorders in patients with long-standing treatment [7], [8].

In this study, we used a multi-contrast approach at 3 T in a population of HIV patients well treated with cART (undetectable viral load) with and without cognitive impairments (MND+ and MND−) as well as a population of healthy sero-negative controls (HC).

The aim of the study was to determine if MND+ patients showed micro-structural brain alterations, changes in myelination integrity and iron deposition compared to MND− and HC. In this context, we tested the null hypotheses that there are no differences: 1) in micro-structural integrity of global white matter (WM) and cortical gray matter (cGM) among MND+, MND− and HC (ii) in micro-structural integrity of sub-cortical nuclei involved in cognitive function (basal ganglia and thalamus) and (iii) in micro-structural integrity of WM and cGM in the frontal, parietal, temporal and occipital lobes.

Lastly, we assessed the null hypothesis that no correlation existed between MRI markers of micro-structural alterations in patients and (i) cognitive signs as well as (ii) HIV infection characteristics.

## Methods

### Subject population

Thirty-six age-matched HIV+ patients with undetectable HIV-1 RNA concentrations (<20 copies/ml for >3 months before study entry) were enrolled: 17 MND+ (53.6±9.1 years, 13 males-M and 4 females-F) and 19 MND- (49±7.2 years, 15 M and 4 F). All participants were enrolled in a randomized pilot study testing the efficacy of rivastigmine on MND and all the MRI were acquired before the beginning of the therapy. The HIV viral load was measured in the cerebrospinal fluid (CSF) of MND+ patients and was undetectable in all of them. We did not examine the CSF of MND− patients because of ethical reasons (absence of complaints and deficits). All patients were treated with cART.

According to the Frascati criteria [9], HIV+ patients were considered as MND+ when they exhibited deficits in ≥2 cognitive domains (performance ≤1 standard deviation below the standardized norms on neuropsychological tests) associated with evidence for mild decreased everyday functioning. Patients with HIV associated-dementia and asymptomatic neurocognitive impairment were not considered for the study. Other exclusion criteria were: (1) history of CNS opportunistic infection, (2) any other opportunistic infection not affecting the brain in the last 12 months before study entry, (3) active drug use, and (4) major depression according to DSM-IV criteria.

Nineteen sero-negative healthy controls (HC: 50±8 years, 9 M and 10 F) were also enrolled in the study. Since none of the HC subjects had cognitive complaints, which has been reported to strongly correlate with normal cognitive functioning in HIV− subjects [10], we only performed a general measure of cognitive function using the mini-mental state examination (MMSE ≥25, HC: 28±2) in this category of study subjects, without additional neuropsychological examination. The study was approved by the Ethics Committee of the Lausanne University Hospital (CHUV) and all subjects gave informed written consent for their participation.

### Neurobehavioral examination in patients

#### Neuropsychological testing

All HIV+ participants underwent a comprehensive neuropsychological evaluation, assessing five cognitive domains generally impaired in HIV+ patients with HIV-associated neurocognitive disorder (HAND) [9], [11]. The functions examined and the applied referenced tests are reported in detail in Table 1. A deficit score for each domain was then calculated using z-scores retrieved from available normative data correcting for age, gender and educational level.

**Table 1:** Neuropsychological tests. References are reported in Data

COGNITIVE TESTS	TESTED FUNCTION	REFERENCES
1. Reaction Time (RTI) from the Cambridge Neuropsychological Test Automated Battery (CANTAB)	speed of information processing	[11], [42], [43]
2. Trail Making Test part A (TMT-A)		
3. Rapid Visual Information Processing [RVIP]	attention/working memory	[11], [42], [43] ,[44]
4. Spatial Working Memory (SWM-Error component) from the CANTAB		
5. Digit spans backward and forward		
6. Trail Making Test part B (TMT-B)	executive functioning	[11], [42], [45] ,[43]
7. Stockings of Cambridge (SOC) from the CANTAB		
8. Spatial Working Memory (SWM-Strategy component) from the CANTAB		
9. Alzheimer’s Disease Assessment Scale-Cognitive Subscale (ADAS-Cog)	verbal learning/memory	[11], [42], [46]
10. Reaction Time (RTI, motor component) from the CANTAB	motor skills	
11. HIV Dementia Scale (HDS) International HDS (IHDS)	screening scales	[11], [42], [47], [48]

#### Psychiatric examination

A psychiatric interview using a French questionnaire (Questionnaire de Santé du Patient) was conducted in order to look for the presence of mood disorders according to the DSM-IV diagnostic criteria. Current mood was assessed using the Hospital Anxiety and Depression scale addressing depressive (HAD-D) and anxious (HAD-A) symptoms, separately. Patients were considered depressed/anxious if the HAD-D/HAD-A subscale score was ≥10/21[11], [12].

#### Functional assessment

The impact of cognitive difficulties on everyday functioning was evaluated through the self-assessment of HIV+ patients. In addition, we conducted the Medical Outcome Study HIV Health Survey (MOS HIV), a questionnaire assessing health-related quality of life (QoL).

### MRI acquisition

**Figure 1:**
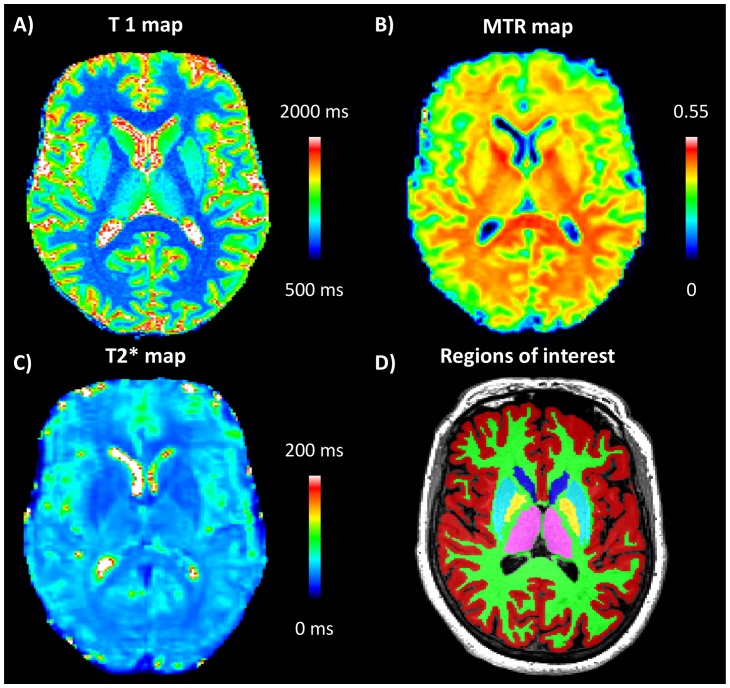
Quantitative/semi-quantitative maps and segmentation in regions of interest. Color coded T1 (A), MTR (B) and T2* (C) maps showing the distribution of the contrasts in an axial slide; In (D), region of interest segmentation of global gray matter (brown), white matter (green), thalamus (pink), caudate (blue), putamen (cyan) and globus pallidus (yellow) in one MND+ subject.

All examinations were carried out on a 3T Magnetom Trio a Tim System (Siemens, Erlangen, Germany) equipped with a 32-channel head coil. A high-resolution T1-weighted Magnetization Prepared Rapid Gradient Echo (MPRAGE) was acquired for anatomical reference (TR/TE  = 2300/2.84 ms, inversion time TI = 900 ms, voxel size  = 1×1×1.2 mm^3^, matrix size  = 256×240×160) [13]. A MP2RAGE acquisition with the same voxel and matrix size was used to assess T1 relaxation maps (TR/TE  = 5000/2.84 ms, inversion times TI1 = 700 ms and TI2 = 2500 ms, FA1 = 4°, FA2 = 5°) [14] (Figure 1 A). Multiple echo Fast Low Angle SHot Magnetic Resonance Imaging (FLASH) with and without magnetization transfer preparation was acquired (TR/TE  = 48/23 ms, voxel size  = 2×2×2 mm^3^, FoV  = 240×256×96, 8 echoes) as described previously [15]. The signals acquired with (MT) and without (M0) the magnetization saturation pulse were used to compute Magnetization Transfer Ratio (MTR) maps, (MTR  =  (M0−MT)/M0*100, Figure 1 B). The 8 echoes of the M0 volumes were used to compute T2 * maps by fitting a mono-exponential decay (Figure 1 C).

Susceptibility Weighted Images (SWI) were acquired in a subset of patients and controls (MND+ n = 14, 53.5±9.3 years, 12 M and 2 F, MND− n = 16, 48±6.4 years, 13 M and 4 F and HC n = 5, 28.6±12.5 years, 3 M and 2 F) using a velocity compensated 3D gradient echo sequence (TR = 50/30 ms, FA  = 18°, voxel size 0.7×0.7×1.4 mm^3^, matrix size  = 180×220×52). Phase images were high-pass filtered to correct for low-frequency phase variations [16].

The MRI protocol (including the SWI sequence) was approved by the ethic committee and performed only after obtaining written consent from all participants.

### Concepts of quantitative and semi-quantitative MRI contrasts

The MTR is a semi-quantitative marker of structural integrity, which is sensitive to the relative proportion of macromolecules (myelin and cellular proteins) and water [17]. A reduced MTR indicates therefore a loss of macromolecules and/or microscopic oedema [17].

Similarly, the quantitative T1 assessment probes micro-structural properties and longer T1 values indicate a loss in tissue structure (macromolecules) [18], [19]. On the other hand, the presence of small molecules with high rotational speed might shorten the T1 relaxation times. In addition, quantitative T1 measurements are biased by local iron presence, with higher levels of iron leading to shorter T1 values [18], [19]. Similarly, T2* relaxation times strongly depend on the local iron content with high iron leading to shortened T2* values [19]. Lastly, the phase information derived from SWI data provides complementary information to the T2* measurement as it is sensitive to myelin alterations and iron accumulation [20].

### Image processing

Bias-field correction and tissue classification were performed on the MPRAGE volumes using an in-house segmentation tool based on a Variational Expectation-Maximization algorithm [21]. The following 6 regions-of-interest (ROI) were extracted in a fully automated fashion: global *white* and *cortical gray matter (WM and cGM)*, *thalamus*, *caudate*, *globus pallidus* and *putamen* (Figure 1 D). Subsequently, T1, MTR and T2* maps were aligned to the MPRAGE volume by a rigid-body registration with 6 degrees of freedom and mutual information cost function using ELASTIX [22]. Rigid and non-rigid registration [23], [24] were performed to align an in-house template (obtained by manual correction of a segmentation provided by Freesurfer software www.surfer.nmr.mgh.harvard.edu in a single healthy subject) to the MPRAGEs volumes. Average MTR, T1 and T2* values were obtained for all ROIs.

In order to minimize registration and partial volume errors: (i) visual inspection and eventual manual correction of deep gray matter nuclei was performed by an experienced neurologist (GC) (ii) a threshold obtained by taking + and −3 standard deviations from the mean value of T1, T2*, MTR histograms in each single ROI in the healthy control group was applied to T1, T2* and MTR values and (iii) morphological erosion was applied on the binary segmentation masks using a disk with 2 voxels radius as structuring element. A 3 voxel radius resulted to be inappropriate for small ROIs like the basal ganglia. In this ways, we tried to verify the influence of possible contaminations from other tissue types. Statistical significance did not change after applying the threshold and the erosion, therefore the original results are reported.

Volumetric information was computed for all the 6 ROIs mentioned above and for WM and cGM of each lobe (frontal, parietal, temporal and occipital). Each volume was normalized by the total intracranial volume (TIV).

SWI data were analyzed using manual delineation of ROIs in the *thalamus*, *caudate, globus pallidus* and *putamen* (ROI size between 143 and 190 mm^3^) in order to avoid susceptibility artifacts at the periphery of these structures, which would have hampered the automatic analysis. Phase differences to CSF (assumed to have zero iron content) were used to assess possibly iron related phase accumulations in each nucleus.

### Statistical analysis

Differences in age, gender and educational levels were assessed using a non-parametric ANOVA (Kruskal-Wallis test) among MND+, MND− and HC. Differences in HIV-infection characteristics (disease duration since diagnosis, duration of aviremia, nadir of CD4 cell count and cART composition) were assessed using Mann Whitney tests between MND+ and MND− and Bonferroni correction for multiple comparisons.

Non parametric ANOVA was used to assess volumetric differences among all the ROIs.

Statistical analysis of parametric MRI data was performed among the 3 groups (MND+, MND− and HC) using permutation-based univariate t-tests (T1, MTR, T2*), bi-variate tests (T1-MTR, MTR-T2* and T1–T2*) as well as multivariate Hotelling tests (T1, MTR and T2*), with 10.000 permutations and age and gender as covariates. Correction for family-wise error rate was performed for multiple comparisons. SWI data were not used for this analysis, because they were available only in a subset of patients and controls.

Multiple regression analysis was performed to assess the influence of MTR, T1, T2* values in all ROIs on global indexes of impairment (cognitive impairment, processing speed, attention, executive, motor and memory index) in MND+ patients. Age and gender were included as covariables.

Similarly, multiple regression analysis was performed to assess the influence of HIV and therapy-dependent characteristics (disease duration, nadir CD4+, aviremia duration, cART duration) on the MTR, T1 and T2* values in all ROIs.

A linear discriminant analysis (LDA) based on T1, MTR and SWI data was performed using the R-software (www.r-project.org). LDA is a statistical procedure to find a linear combination of features that characterizes or separates two or more classes of objects and events [25]. A leave-one out test was performed for cross-validation of prediction.

## Results

HIV + patients, either MND+ or MND−, did not differ from HC in age, education and gender (p>0.3, 0.3 and 0.07, respectively).

MND+ and MND− groups did not differ in age, gender, educational level and HIV-characteristics (disease duration since diagnosis, duration of aviremia, current CD4 T-cells count, nadir CD4, cART composition) (Table 2). In particular, there was a similarly high level of CD4+ T cells (>600/µl) and a CPE greater than 7 in both categories, indicating that both MND+ and MND− were immunocompetent and optimally treated. Furthermore, there was no difference with regard to potentially neurotoxic drugs (see Table S1).

**Table 2:** Demographics, HIV characteristics and neuropsychological results (raw scores and indexes) of MND+ and MND− patients.

	MND+ (n = 17)	MND- (n = 19)	p-value
Age, y	53.6±9.1	49±7.2	0.1
Gender, men (%)	13 (76)	16 (84)	0.6
Education (≥ secondary school), n (%)	12 (71)	18 (95)	0.1
Duration of HIV infection, y	13.6±7.3	14.7±6.7	0.7
Duration of HIV aviremia, y	4.9±3.5	6.4±3.7	0.2
CD4^+^/μl	648.8±217.3	647.1±341.3	0.5
Nadir CD4^+^/μl	165.4±88.7	198.4±124	0.6
CPE score for current cART	7.6±1.9	7.3±1.7	0.7
HDS score	9.4±3.8	14.1±2.8	<0.001
IHDS score	9±2.1	10.8±1.4	<0.01
ADAS-Cog	7.1±2.9	3.2±1.3	<0.001
Global cognitive index	15.6±4.8	3.7±1.7	<0.0001
Processing speed index	3.3±1.8	1.3±1	<0.01
Attention/working memory index	6.8±2.4	1.4±1.2	<0.0001
Executive index	3.1±1.2	0.7±0.7	<0.0001
Memory index	1.6±1.1	0.1±0.3	<0.0001
Motor index	0.8±1.1	0.2±0.4	0.1

Yet, MND+ patients showed significantly lower scores in all cognitive indexes than MND− patients after Bonferroni correction for multiple comparisons (Table 2).

### MRI analysis

**Figure 2:**
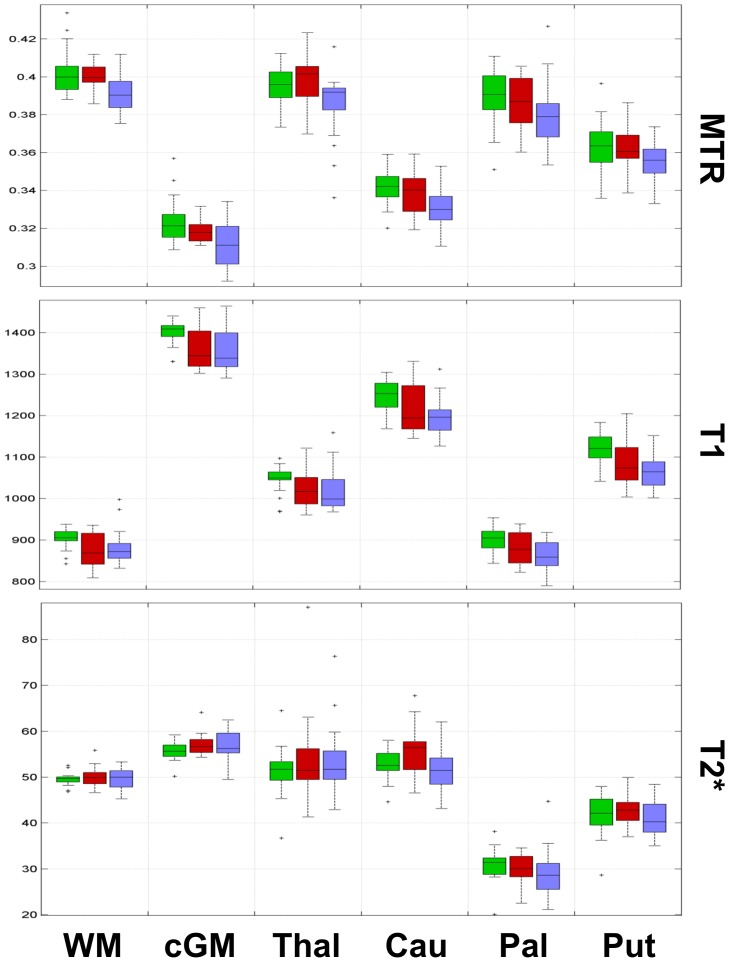
Boxplots representing data from the univariate analysis for WM, cGM, thalamus and basal ganglia. In each box, the central mark represents the median, the edges represent the 25th and 75th percentiles and the whiskers extend to the most extreme data points not considered outliers; outliers are plotted individually as x. HC: green; MND−: red; MND+: blue.

**Figure 3:**
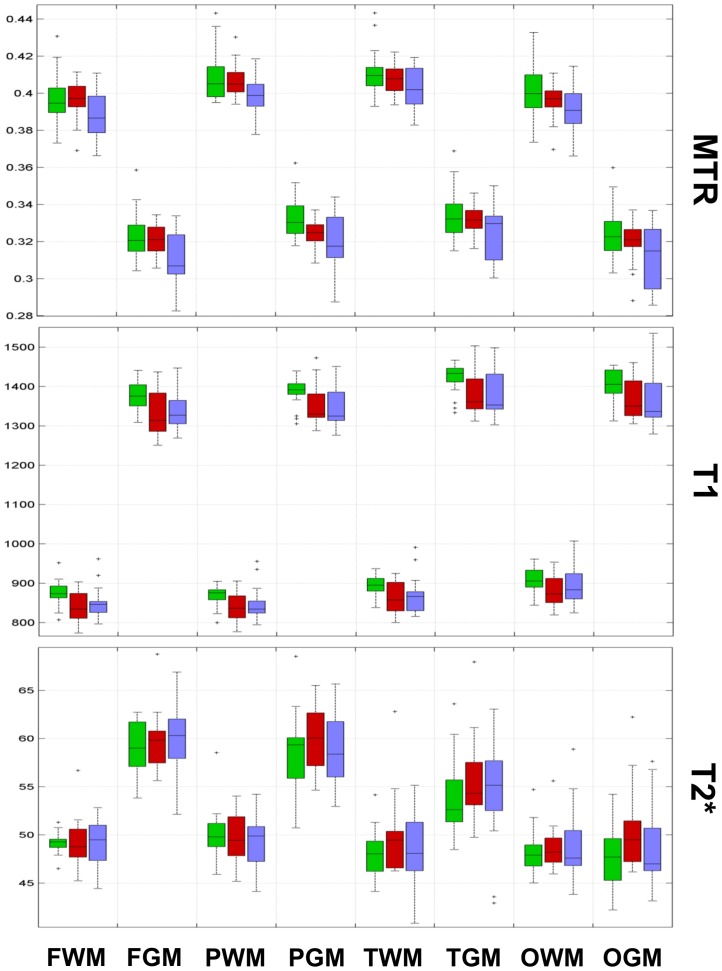
Boxplots representing data from the univariate analysis for lobar WM and cGM. In each box, the central mark represents the median, the edges represent the 25th and 75th percentiles and the whiskers extend to the most extreme data points not considered outliers; outliers are plotted individually as x. HC: green; MND−: red; MND+: blue. FWM: frontal WM; PWM: parietal WM; TWM: temporal WM; OWM: occipital WM; FcGM: frontal cGM; PcGM: parietal cGM; TcGM: temporal cGM; OcGM: occipital cGM.

**Figure 4:**
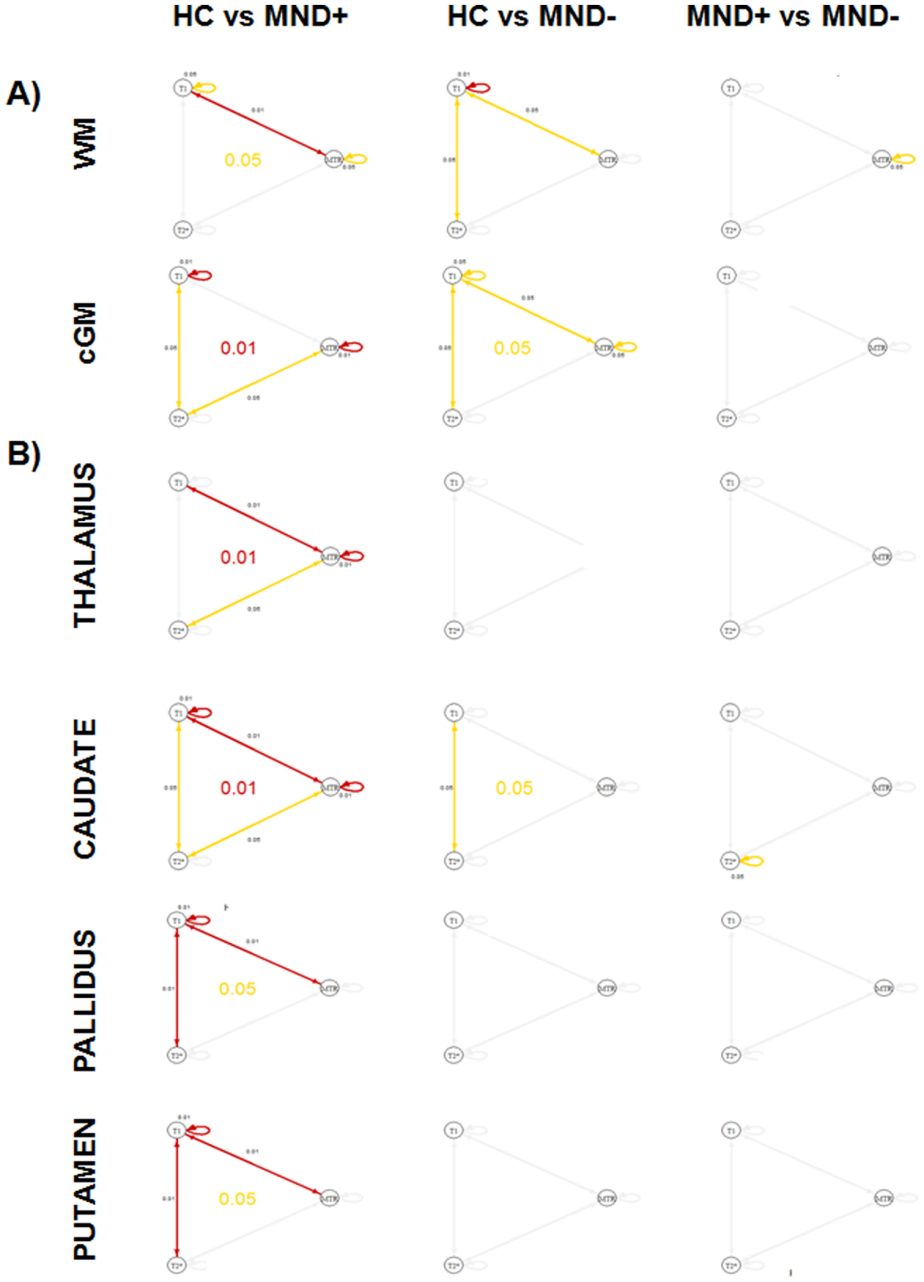
Graphs showing the respective significance in each ROI for univariate analysis (loop), bivariate analysis (straight line) and multivariate analysis (central circle). Red color indicates p≤0.01 and yellow color indicates p≤0.05. A) hypothesis 1, B) hypothesis 2.

**Figure 5:**
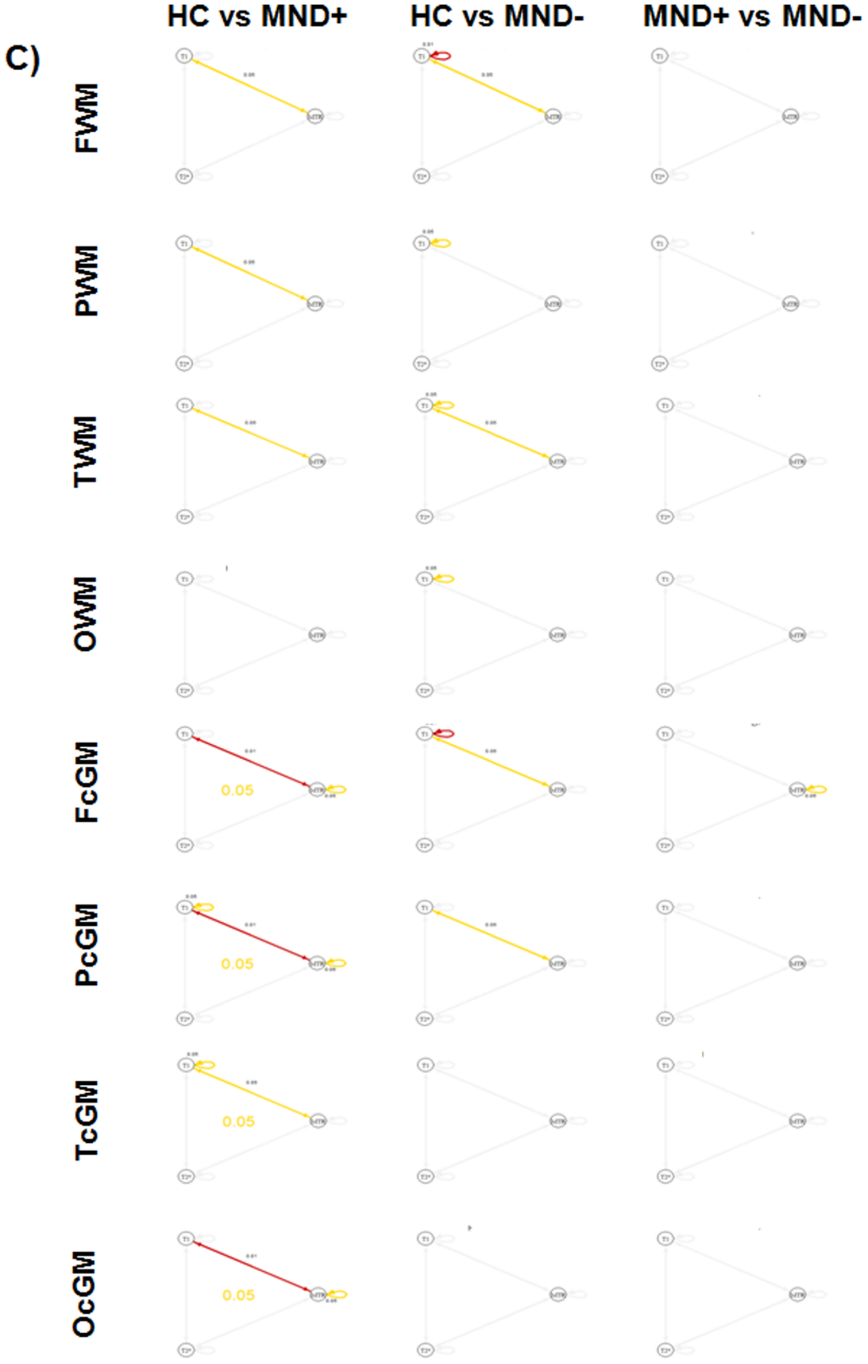
Graphs showing the respective significance in each ROI for univariate analysis (loop), bivariate analysis (straight line) and multivariate analysis (central circle). Red color indicates p≤0.01 and yellow color indicates p≤0.05. Hypothesis 3.

Figure 2 and 3 shows the results of the comparison among groups as well as their statistical significance (figures 4 and 5).

Univariate analysis showed (i) lower MTR in WM, cGM, thalamus and caudate as well as (ii) lower T1 in all regions but the thalamus when MND+ patients where compared to HC. The same analysis showed (i) lower MTR in cGM and (ii) lower T1 in WM and cGM of MND− patients vs HC. The univariate comparison between MND+ and MND− patients revealed additionally lower MTR in WM of MND+ subjects and higher T2* in the caudate of MND− patients (figures 2, 3, 4, and 5).

In addition, bivariate and multivariate analysis showed that combination of contrasts revealed significant differences in all regions for MND+ patients vs controls as well as in WM, cGM and caudate of MND− patients vs controls (figures 4 and 5).

No lobar predominance of WM and cGM changes was observed (figures 3 and 5).

In summary, therefore, our data refute the 3 null hypotheses.

No volumetric differences were seen among groups in WM, cGM, sub-cortical nuclei and lobar cgM and WM (see Table S2).

### Multiple regression analyses between MRI markers and neuropsychological indexes in MND+ patients

We found that T1, T2* and MTR values in the sub-cortical nuclei were correlated with the executive index in MND+ patients (p = 0.01 and multiple R^2^ = 0.95, adjusted R^2^ = 0.85). In particular, the parameters that were mostly correlated were the MTR in the putamen (p = 0.05) and the T1 in globus pallidus and putamen (p = 0.03). Age and gender did not appear to play any significant effects on the executive index (p>0.5).

### Multiple regression analyses between MRI markers and biological variables in HIV+ patients

We found that biological variables related to HIV infection were not correlated with MRI markers of disease.

### Linear discriminant analysis to differentiate between MND+ and MND- based on MRI data

The linear discriminant analysis based on T1, MTR and SWI data distinguished MND + from MND-patients with a classification quality of 73% after cross-validation (specificity  = 76.5% and sensitivity  = 69%). T2* data did not add any significance to the model.

## Discussion

Our study shows the presence of diffuse micro-structural brain tissue alterations in MND+ and MND− HIV+ patients compared to a population of HIV− healthy controls, despite effective treatment and undetectable viremia and virorachia. Most of the observed alterations were more pronounced in MND+ than in MND− patients and were significantly more severe in global WM.

### Biological substrate of MTR, T1 and T2* changes

We provide evidence of a significant decrease of MTR in MND+ patients compared to HC in WM, cGM, thalamus and caudate, whereas MND− patients show a less prominent decrease of MTR in cGM (Figures 2 and 4 A–B). Lower MTR indicates a loss of macromolecules (myelin and cellular proteins) and/or a micro-oedema effect [26], [27], phenomena that are consistent with an on-going inflammation triggered by HIV [28].

On the other hand, we observe longer T2* relaxation times in the caudate of MND− patients compared to MND+ patients; the same patter was observed when MND− patients were compared to HC but it did not reach significance (Figures 2 and 4 B). . Whether this aspect plays a role in the pathophysiology of MND and/or in the development of HIV infection and response to cART should be determined in prospective studies.

As to the T1 contrast, its behaviour is complex; based on the MTR and T2* behaviour, we would have expected a general increase in T1 in HIV+ patients vs. HC. However, T1 relaxation times in MND− and MND+ patients are generally shorter than HC (Figures 2, 3, 4, and 5), a behaviour that might reflect lower iron content (above all in the presence of longer T2*) and/or the presence of small non-paramagnetic molecules with high rotational speed (tissue debris, cART compounds, etc) [18], [29] that would not influence T2* relaxation times.

### Clinical implication of MTR, T1 and T2* changes in aviremic HIV+ patients

Previous clinical studies reported that cognitive impairment in HIV-infected individuals can occur in the context of maximal viral suppression in the serum [30]–[33]. Some of these studies showed a correlation between cognitive deficits and age [31], [32]. Other works suggested that cognitive impairment may be an effect of HIV-induced accelerated aging [34] or neurodegenerative processes dependent on chronic inflammation [35]. Recent studies additionally proposed that insufficient penetration of cART in the CNS [6] or, on the contrary, a drug-dependent toxic effect [8] might play a role in MND physiopathology in aviremic HIV+ patients.

Nevertheless, MND+ and MND− patients enrolled in our study did not differ in terms of age, gender, nor for any HIV-related characteristics (undetectable plasma HIV viral load in all, current CD4+ T cell count, CD4+ nadir, duration of aviremia, cART CPE score[6] as well as composition and duration). Furthermore, the HIV viral load in the CSF was negative in all MND+ patients, suggesting that there was no overt HIV replication in the CNS.

In this context, therefore, neither current HIV infection nor drug response or toxicity appears to be a clear cause of the presence of MND.

Thus, how to explain that some of these well-treated HIV+ patients develop MND and other not? We show that the micro-structural differences between HC and MND+ are located in regions particularly prone to HIV infection (WM and basal ganglia) [30] as well as in multiple lobes of the cerebral cortex, which have been previously shown to be thinner in HIV+ patients with cognitive impairment [31], [32]. Thus, despite the fact that there is no difference in terms of HIV characteristics between MND+ and MND−, we hypothesize that, upon HIV infection, some patients are less able than others to control HIV replication or to limit its collateral damages. This relative inefficiency will lead to chronic low grade inflammation and tissue damage causing cognitive disorders.

In favour of this hypothesis, we point to the strong correlation between MRI results in the sub-cortical gray matter nuclei (putamen and globus pallidus) and executive impairment. Deficits in executive function have been largely described in untreated HIV+ patients [9], [36] as a consequence of HIV infection in the basal ganglia and deep white matter. And previous studies showed that fronto-striatal circuits connecting the lateral-prefrontal cortex to the caudate and glubus pallidus play a major role in executive functions (for review see [37]), and the putamen has been recently reported to be implicated in working memory processes [38].

To date, there are no post-mortem studies reporting chronic inflammation markers in the brain of HIV+ patients under effective cART; the few studies available on treated HIV+ patients did not include aviremic subjects and reported aspecific brain pathology, which did not relate to premortem neurocognitive deficits [39], [40]. Future works should help elucidate the presence of subtle chronic inflammatory processes and/or neurodegeneration in the brain of well-treated HIV+ patients with MND.

### Potential of multi-parametric MRI to study subtle brain abnormalities

Previous MRI studies reported volumetric and morphometric changes of the whole brain, basal ganglia and white matter in HIV patients that correlated with cognitive symptoms (for review see [41]). Notwithstanding, none of these previous works specifically studied MND+ patients under effective cART and with undetectable viral load. Our study does and shows that multi-parametric MRI at 3T is more sensitive than volumetric analysis to investigate the presence and the nature of subtle abnormalities.

Last, another important finding of our study is that a linear discriminant analysis based on T1, MTR and SWI data shows that multi-parametric MRI provides the capability to distinguish between MND+ and MND− patients, even when both categories are optimally treated, reaching classification quality of 73% after cross-validation. This result is particularly important as it evidences the potential future role of new clinically-compatible MRI approached to support diagnostic processes in the absence of neuropsychological expertise as well as to monitor response to therapy and disease evolution.

In summary, multi-parametric MRI at high field appears a powerful means to investigate the physiopathology of minor neurological signs and a promising sensitive tool to support medical diagnosis and follow-up. Future validation of these methods is planned on a prospective study in Lausanne neuro-HIV cohort.

## Supporting Information

Table S1 **Presence of potentially neurotoxic drugs (in %) in MND+ and MND− patients.**
(DOCX)

Table S2 **ROIs volume in mm3.**
(DOCX)

Data S1 **Supplementary data.**
(DOCX)
