# Supplementary material for: Micro-Structural Brain Alterations in Aviremic HIV+ Patients with Minor Neurocognitive Disorders: A Multi-Contrast Study at High Field
Source: PLoS One. 2013 Sep 10;8(9):e72547. doi: 10.1371/journal.pone.0072547 (PMC3769352; doi:10.1371/journal.pone.0072547)
Supplement: Table S1 — Presence of potentially neurotoxic drugs (in %) in MND+ and MND− patients. (DOCX) [file pone.0072547.s001.docx]

**Table S1:**

|  | MND+ | MND- |
| --- | --- | --- |
| Efavirenz (EFV): | 24 (%) | 32 (%) |
| Tenofovir (TDF): | 71 (%) | 53 (%) |
| Zidovudin (AZT) | 6 (%) | 21 (%) |
| Emtricitabine (FTC) | 53 (%) | 37 (%) |
| Didanosine (DDI) | 0 | 0 |
