# Supplementary material for: Micro-Structural Brain Alterations in Aviremic HIV+ Patients with Minor Neurocognitive Disorders: A Multi-Contrast Study at High Field
Source: PLoS One. 2013 Sep 10;8(9):e72547. doi: 10.1371/journal.pone.0072547 (PMC3769352; doi:10.1371/journal.pone.0072547)
Supplement: Table S2 — ROIs volume in mm3. (DOCX) [file pone.0072547.s002.docx]

**Table S2:**

| **SUBJECT** | **GM** | **WM** | **RThalamus** | **LThalamus** | **RCaudate** | **LCaudate** | **RPutamen** | **LPutamen** | **RPallidum** | **LPallidum** |
| --- | --- | --- | --- | --- | --- | --- | --- | --- | --- | --- |
| C | 0.430836908 | 0.380811775 | 0.004977858 | 0.004805136 | 0.002992912 | 0.002695967 | 0.003608381 | 0.003833813 | 0.001407076 | 0.001453171 |
| C | 0.407787035 | 0.372911355 | 0.005105147 | 0.004985831 | 0.002540864 | 0.002817914 | 0.003106273 | 0.003279304 | 0.001094019 | 0.001269347 |
| C | 0.445852283 | 0.360038744 | 0.005430106 | 0.005282655 | 0.002920794 | 0.002957954 | 0.003731597 | 0.004258111 | 0.001235862 | 0.001516549 |
| C | 0.429234663 | 0.363776668 | 0.005642356 | 0.00526693 | 0.002811517 | 0.002784306 | 0.003570296 | 0.003826605 | 0.001210962 | 0.001386738 |
| C | 0.43546233 | 0.348343667 | 0.005610995 | 0.00539218 | 0.00281319 | 0.002572321 | 0.003470723 | 0.00385965 | 0.001316017 | 0.001562588 |
| C | 0.43533003 | 0.36728022 | 0.005254045 | 0.005361008 | 0.002931216 | 0.003101268 | 0.004077198 | 0.003904359 | 0.001272984 | 0.001313732 |
| C | 0.476912143 | 0.3665458 | 0.005234488 | 0.005199755 | 0.003116516 | 0.003121793 | 0.004015652 | 0.004102476 | 0.00117329 | 0.001359447 |
| C | 0.465531278 | 0.35415425 | 0.00504043 | 0.004773509 | 0.002923344 | 0.002899503 | 0.00399865 | 0.003906468 | 0.001239447 | 0.001196175 |
| C | 0.442288996 | 0.361157154 | 0.005659785 | 0.005540079 | 0.003321329 | 0.003150761 | 0.003848999 | 0.003598799 | 0.001181621 | 0.001340007 |
| C | 0.446461436 | 0.372192967 | 0.004388438 | 0.004517739 | 0.002851629 | 0.002937058 | 0.004240432 | 0.004144948 | 0.001547696 | 0.001656198 |
| C | 0.437224574 | 0.378603958 | 0.004507723 | 0.004405301 | 0.002977807 | 0.002659962 | 0.004089141 | 0.004146112 | 0.001406973 | 0.001384391 |
| C | 0.464011321 | 0.373122683 | 0.00453165 | 0.004476429 | 0.002650316 | 0.002340502 | 0.00409063 | 0.004151805 | 0.001335261 | 0.001510194 |
| C | 0.432296979 | 0.36275269 | 0.004538359 | 0.004665695 | 0.003013245 | 0.00301129 | 0.004033257 | 0.004042229 | 0.001228673 | 0.0014944 |
| C | 0.431978555 | 0.360564955 | 0.004591989 | 0.004404367 | 0.002948649 | 0.002833952 | 0.003726796 | 0.003927097 | 0.001141662 | 0.001390368 |
| C | 0.43494905 | 0.373488876 | 0.004799184 | 0.004978462 | 0.002911701 | 0.002831925 | 0.00423789 | 0.004490281 | 0.001464938 | 0.001615611 |
| C | 0.458516069 | 0.352939568 | 0.005794957 | 0.005495093 | 0.003547399 | 0.003131124 | 0.004120543 | 0.004254855 | 0.001324313 | 0.001632007 |
| C | 0.415434819 | 0.381448631 | 0.004975175 | 0.004763162 | 0.003084153 | 0.00292064 | 0.003873757 | 0.004061188 | 0.00128236 | 0.0015276 |
| C | 0.432408493 | 0.357221274 | 0.005373772 | 0.00517621 | 0.002921862 | 0.0030729 | 0.003703224 | 0.003943217 | 0.001164572 | 0.001273902 |
| C | 0.439743354 | 0.369163093 | 0.005578573 | 0.005407922 | 0.002812551 | 0.002781854 | 0.00358778 | 0.003996236 | 0.001092844 | 0.001596312 |
| C | 0.430693946 | 0.369428853 | 0.005556344 | 0.00506134 | 0.003384849 | 0.00307231 | 0.003822755 | 0.003865218 | 0.001296221 | 0.001578075 |
| MND- | 0.450151239 | 0.35092145 | 0.004724569 | 0.00466342 | 0.002542002 | 0.002530591 | 0.003379981 | 0.003661572 | 0.001057835 | 0.001429084 |
| MND- | 0.453887476 | 0.368680415 | 0.005589568 | 0.004716054 | 0.003228281 | 0.002966666 | 0.004193351 | 0.004817909 | 0.001274999 | 0.001715472 |
| MND- | 0.446870919 | 0.361572032 | 0.005020859 | 0.004949905 | 0.002556223 | 0.002693489 | 0.003338244 | 0.003726162 | 0.00115066 | 0.001279252 |
| MND- | 0.43998952 | 0.367659533 | 0.005057702 | 0.005097945 | 0.002747528 | 0.002663341 | 0.003875348 | 0.003902975 | 0.001209628 | 0.001466531 |
| MND- | 0.442013867 | 0.337797832 | 0.004815639 | 0.004620269 | 0.002833535 | 0.002630563 | 0.003699179 | 0.003754737 | 0.001281288 | 0.001426438 |
| MND- | 0.437413218 | 0.38031939 | 0.004968146 | 0.004857284 | 0.003107618 | 0.002813698 | 0.003849605 | 0.00397674 | 0.00125787 | 0.001520457 |
| MND- | 0.413230382 | 0.367679881 | 0.005170919 | 0.005162734 | 0.003156979 | 0.003009656 | 0.003365798 | 0.003742921 | 0.001183479 | 0.001341357 |
| MND- | 0.417573358 | 0.379354813 | 0.005041816 | 0.004601622 | 0.002682538 | 0.002872532 | 0.003494691 | 0.003483129 | 0.001162857 | 0.001113464 |
| MND- | 0.435068624 | 0.379730741 | 0.004981275 | 0.004824007 | 0.002962456 | 0.002905623 | 0.004074093 | 0.004218633 | 0.001353249 | 0.001533471 |
| MND- | 0.404395809 | 0.39409416 | 0.004456628 | 0.004268501 | 0.00304831 | 0.00278449 | 0.004286703 | 0.004308196 | 0.001370687 | 0.001543053 |
| MND- | 0.415883397 | 0.374333477 | 0.005436922 | 0.004978914 | 0.002486467 | 0.002393157 | 0.003784861 | 0.003931162 | 0.001284032 | 0.001600106 |
| MND- | 0.431067157 | 0.352724956 | 0.004935759 | 0.005072255 | 0.002635333 | 0.002443028 | 0.003953126 | 0.003705875 | 0.001128043 | 0.001378887 |
| MND- | 0.445488391 | 0.375334578 | 0.004900675 | 0.00490133 | 0.002814645 | 0.002565317 | 0.003431868 | 0.00357783 | 0.001056459 | 0.001232299 |
| MND- | 0.40237209 | 0.380772519 | 0.00510537 | 0.00505129 | 0.002862352 | 0.002730168 | 0.003691803 | 0.003889194 | 0.001118058 | 0.001232116 |
| MND- | 0.441658294 | 0.368249946 | 0.005771842 | 0.005824278 | 0.003163233 | 0.003115113 | 0.003247428 | 0.003546812 | 0.001348916 | 0.001467935 |
| MND- | 0.447452827 | 0.374945545 | 0.00435767 | 0.004076655 | 0.002755239 | 0.002655888 | 0.00359559 | 0.003753768 | 0.001176922 | 0.001396759 |
| MND- | 0.446954173 | 0.379193516 | 0.004794683 | 0.004365026 | 0.002753392 | 0.002678587 | 0.00379399 | 0.00390615 | 0.001328157 | 0.001326276 |
| MND- | 0.441476221 | 0.350847247 | 0.004804212 | 0.004809986 | 0.002761937 | 0.002755001 | 0.003499117 | 0.003394856 | 0.001111597 | 0.001186298 |
| MND- | 0.443168124 | 0.361159763 | 0.006280878 | 0.005952588 | 0.003443489 | 0.003019431 | 0.003719892 | 0.004026135 | 0.001122933 | 0.001456599 |
| MND+ | 0.436197549 | 0.364912648 | 0.004935567 | 0.004916522 | 0.002991714 | 0.003190399 | 0.003236481 | 0.003799637 | 0.001041968 | 0.001272668 |
| MND+ | 0.42668332 | 0.381791356 | 0.004515668 | 0.004644566 | 0.0025457 | 0.002373503 | 0.003540507 | 0.003596077 | 0.00113021 | 0.00110971 |
| MND+ | 0.427492758 | 0.364209402 | 0.005089158 | 0.005136205 | 0.0027487 | 0.002607329 | 0.004130523 | 0.003888807 | 0.001406179 | 0.001533283 |
| MND+ | 0.447701937 | 0.369607074 | 0.004379173 | 0.004457376 | 0.002943639 | 0.002777006 | 0.004145447 | 0.004243318 | 0.001336206 | 0.001503127 |
| MND+ | 0.41895222 | 0.390762664 | 0.005734437 | 0.004766513 | 0.003475359 | 0.002832122 | 0.004116722 | 0.004168654 | 0.001439895 | 0.001596247 |
| MND+ | 0.445050983 | 0.384048409 | 0.005097923 | 0.004843328 | 0.003010873 | 0.002862002 | 0.003973442 | 0.003844093 | 0.001280201 | 0.001400756 |
| MND+ | 0.434842503 | 0.363316071 | 0.0052038 | 0.005098529 | 0.002677224 | 0.00290615 | 0.004116084 | 0.00427655 | 0.001373548 | 0.001660999 |
| MND+ | 0.427835002 | 0.338950577 | 0.005683242 | 0.005296146 | 0.002807908 | 0.002781015 | 0.003400236 | 0.003230686 | 0.001209328 | 0.00131053 |
| MND+ | 0.466925972 | 0.341697097 | 0.005247866 | 0.0049636 | 0.002675248 | 0.002492913 | 0.003395173 | 0.00348444 | 0.001060475 | 0.001264199 |
| MND+ | 0.428473035 | 0.369362577 | 0.004520981 | 0.004284824 | 0.0027406 | 0.002752201 | 0.003887464 | 0.004268297 | 0.001293584 | 0.00136431 |
| MND+ | 0.456046861 | 0.366044998 | 0.005447764 | 0.005299712 | 0.002903707 | 0.00258749 | 0.004013244 | 0.00383418 | 0.001263044 | 0.001349073 |
| MND+ | 0.419595325 | 0.396588268 | 0.004660895 | 0.004503957 | 0.002809387 | 0.002711805 | 0.00357338 | 0.003541566 | 0.001171908 | 0.001256393 |
| MND+ | 0.425432598 | 0.37776822 | 0.006175583 | 0.005872689 | 0.003688362 | 0.003305156 | 0.003774618 | 0.004015641 | 0.001278285 | 0.001476491 |
| MND+ | 0.445697815 | 0.373501655 | 0.004133186 | 0.004345495 | 0.002419663 | 0.002340365 | 0.003476355 | 0.003653731 | 0.001097952 | 0.001412087 |
| MND+ | 0.407189653 | 0.346176712 | 0.005394447 | 0.005262677 | 0.003419493 | 0.003201731 | 0.004605579 | 0.004120682 | 0.001250245 | 0.001349808 |
| MND+ | 0.409410834 | 0.371779996 | 0.005309465 | 0.005162372 | 0.002982694 | 0.00314619 | 0.003807118 | 0.003786779 | 0.001280528 | 0.001452631 |
| MND+ | 0.395430006 | 0.342529968 | 0.005344681 | 0.005162374 | 0.00302044 | 0.003195183 | 0.003296948 | 0.002990656 | 0.00101666 | 0.001007137 |
| MND+ | 0.439429558 | 0.358589931 | 0.005320425 | 0.005552845 | 0.002439639 | 0.002634146 | 0.003417684 | 0.003391955 | 0.001028115 | 0.001287496 |
|  |  |  |  |  |  |  |  |  |  |  |
| T-test HC vs MND- | 0.33043634 | 0.525831913 | 0.652506098 | 0.402010232 | 0.203516335 | 0.04469147 | 0.120030843 | 0.212579147 | 0.09239988 | 0.281480801 |
| T-test HC vs MND+ | 0.095207962 | 0.662552881 | 0.975307571 | 0.911371615 | 0.647187764 | 0.485580396 | 0.572319836 | 0.087151996 | 0.430784324 | 0.091718571 |
| T-test MND- vs MND- | 0.131788859 | 0.918114556 | 0.961879855 | 0.868428269 | 0.484927541 | 0.420740912 | 0.498760764 | 0.071729467 | 0.226222641 | 0.087293152 |
